# Mitochondrial Genome Sequence of the Glass Sponge *Oopsacas minuta*

**DOI:** 10.1128/genomeA.00823-15

**Published:** 2015-07-30

**Authors:** Cyril Jourda, Sébastien Santini, Caroline Rocher, André Le Bivic, Jean-Michel Claverie

**Affiliations:** a Aix-Marseille Université, CNRS UMR 7256 (IMM FR 3479), Structural and Genomic Information Laboratory, Marseille, France; b Aix-Marseille Université, CNRS UMR, Mediterranean Institute of Biodiversity and Ecology Marine and Continental (IMBE), Station Marine d’Endoume, Marseille, France; c Aix-Marseille Université, CNRS UMR, Developmental Biology Institute of Marseille Luminy (IBDM), Marseille, France; d Assistance Publique des Hopitaux de Marseille, Marseille, France

## Abstract

We report the complete mitochondrial genome sequence of the Mediterranean glass sponge *Oopsacas minuta*. This 19-kb mitochondrial genome has 24 noncoding genes (22 tRNAs and 2 rRNAs) and 14 protein-encoding genes coding for 11 subunits of respiratory chain complexes and 3 ATP synthase subunits.

## GENOME ANNOUNCEMENT

Sponges (*Porifera* phylum) comprise a sister group of “true” animals (Eumetazoa) divided in four major classes and representing a key group to retrace the early evolution of animals. The phylogeny positions of sponges are still unclear, and mitochondrial protein-coding genes may be of help in resolving standing evolutionary issues, as well as in population/ecological studies (1). *Oopsacas minuta* is a small white glass sponge (*Hexactinellida* class, *Lyssacinosida* order) with a siliceous skeleton. Discovered in the straits of Gibraltar, this sponge is distributed only in the Mediterranean Sea in both deep water and caves (2). Little information was available until eight mitochondrial DNA (mtDNA) sequences of glass sponges (including a nearly complete mtDNA sequence from *Oopsacas minuta*) were recently reported but not yet available in GenBank (3). Here, we present the complete mtDNA sequence of *Oopsacas minuta*, the first complete mtDNA sequence of a *Lyssacinosida* representative released in a database.

Recently, we sequenced the draft genome of *Oopsacas minuta* using Illumina technology with DNA-seq paired-end and Nextera mate pair protocols on a HiSeq2500 sequencer. Adapter sequences were removed and low-quality bases (threshold of 28) of reads were trimmed using Cutadapt (4). After cleaning, 10% of the 395,851,746 paired-end reads were picked randomly and assembled into 50,958 contigs using IDBA-UD (5). Contigs were scaffolded into 38,533 sequences with the scaffolding tool of the Platanus assembler (6) using the complete data set of clean Illumina reads. The scaffold corresponding to the mtDNA was identified based on the homology search using the partial mtDNA sequence (EF537577.1) of *Sympagella nux*, another glass sponge of the order *Lyssacinosida*. The assembly was checked using read-mapping information of insert sizes and orientation of paired-ends mapped onto mtDNA assembly with Bowtie version 1.1.1 (7). The coverage of the mtDNA sequence was estimated at 1,448 based on Bowtie results using SAMtools (8).

The complete mtDNA sequence is 19 kbp long with a G+C content of 31%. Comparison with mtDNA sequences available in GenBank showed that *Sympagella nux* (score, 8,587), *Iphiteon panicea* (score, 5,439), *Aphrocallistes vastus* (score, 1,477), *Lophophysema eversa* (score, 1,197), and *Bolosoma* sp. USNM 1097546 (score, 1,184), all glass sponges, were the closest neighbors of *Oopsacas minuta*. The structural and functional annotation was performed with the mitochondrial genome annotation (MITOS) server (9) and the ARWEN program (10). Annotations of genes were checked using homology searches on GenBank and eventually improved using Artemis (11). A single DNA strand carried genes with a total of 22 tRNA genes, 2 rRNA genes (one large and one small rRNA subunit), and 14 protein-coding genes. Non-protein-coding genes and coding sequences represented 20% and 62.5% of the genome, respectively. This genome has 13 of the respiratory genes common to most animal mtDNA (*atp6*, *atp8*, *cob*, *cox1*, *cox2*, *cox3*, *nad1*, *nad2*, *nad3*, *nad4*, *nad4l*, *nad5*, and *nad6*) and the *atp9* gene found in nearly all sponge mtDNA. No *trnaW* gene was identified, suggesting a lineage-specific gene loss in the mtDNA sequence of *Oopsacas minuta*. No occurrence of the +1 frameshifting previously reported in other glass sponge mitochondria was observed.

### Nucleotide sequence accession numbers.

The mtDNA sequence of *Oopsacas minuta* is available at DDBJ/EMBL/GenBank under the accession number KR709158. The version described in this paper is the first version, KR709158.1.
